# Volatile Composition and Sensory Properties of Mead

**DOI:** 10.3390/microorganisms7100404

**Published:** 2019-09-29

**Authors:** Ana Paula Pereira, Ana Mendes-Ferreira, Luís G. Dias, José M. Oliveira, Leticia M. Estevinho, Arlete Mendes-Faia

**Affiliations:** 1 WM&B—Wine Microbiology & Biotechnology Lab, Department of Biology and Environment, University of Trás-os-Montes and Alto Douro, 5001-801 Vila Real, Portugal; appereira@ipb.pt (A.P.P.); anamf@utad.pt (A.M.-F.); afaia@utad.pt (A.M.-F.); 2 Department of Biology and Biotechnology, Agricultural College of Bragança, Polytechnic Institute of Bragança, Campus Santa Apolónia, 5300-253 Bragança, Portugal; ldias@ipb.pt; 3 Mountain Research Center (CIMO), Polytechnic Institute of Bragança, Campus Santa Apolónia, 5300-253 Bragança, Portugal; 4 Biosystems & Integrative Sciences Institute, Faculty of Sciences University of Lisbon, 1749-016 Lisboa, Portugal; 5 CEB−Centre of Biological Engineering, University of Minho, 4710-057 Braga, Portugal; jmoliveira@deb.uminho.pt

**Keywords:** aroma volatile compounds, mead, sensory analysis

## Abstract

Mead is a traditional beverage that results from the alcoholic fermentation of diluted honey performed by yeasts. Although the process of mead production has been optimized in recent years, studies focused on its sensory properties are still scarce. Therefore, the aim of this work was to analyse the sensory attributes of mead produced with free or immobilized cells of the *Saccharomyces cerevisiae* strains QA23 and ICV D47, and to establish potential correlations with its volatile composition. In the volatile composition of mead, the effect of yeast condition was more important than the strain. In respect to sensory analysis, the most pleasant aroma descriptors were correlated with mead obtained with free yeast cells, independently of the strain. Both sensory analysis and volatile composition indicates that the most pleasant mead was produced by free yeast cells. Although this study has provided a significant contribution, further research on the sensory quality of mead is still needed.

## 1. Introduction

Volatile aroma compounds play a key role in the quality of alcoholic beverages being the main compounds responsible for aroma and mouthfeel perception [1]. Concerning wine, its aroma is composed by the varietal aroma that arises directly from grapes with minor modifications; fermentative aroma compounds, produced by yeasts during the alcoholic fermentation; and the maturation bouquet that results from chemical reactions during storage and ageing [2,3,4,5]. Regarding mead aroma, it has contributions from honey, the yeast used for inoculation and fermentation conditions [6,7]. Indeed, alcoholic fermentation increases the number and concentration of the volatiles and enhances the aroma properties leading to a characteristic, aromatic and healthy beverage. The combination of volatile compounds defines the quality of the beverage and therefore allows the distinction between them [8].

The quality of food and beverages can be evaluated, essentially, by subjective or objective methods. An example of an objective analysis technique is the identification and quantification of aroma volatile compounds by gas chromatography (GC). Nevertheless, to assess the contribution of individual compounds to the overall odour of a beverage, it is important to determine odour activity values (OAVs), which correspond to the concentration of a single compound divided by its odour perception threshold. In general, aroma active compounds are volatiles which have concentration in the beverage that is above their perception threshold (i.e., OAV > 1), yet it is necessary to take into account the synergistic/additive effects between compounds [5].

Chemical analysis by itself can not completely define and evaluate the quality of a food product. Thus, subjective methods based on human assessment, such as sensory analysis are almost always indispensable in identifying significant sensory attributes of food quality, food flavour characteristics and consumer preferences [9,10]. Overall, the most important sensory characteristics of beverages are evaluated through smell, taste and, to a lesser extent, colour and appearance [3] among other visual proprieties, and are performed by a panel of experts or consumers. However, there is considerable agreement that the sensory perception of any beverage is highly variable because it can be influenced by individual preferences; even between tasters of an experienced and trained panel of experts [10].

The correlation between chemical and sensory data is already being exploited for definition of wine quality [8,11]. This approach can lead to a better understanding of wine or other food product characteristics [9].

Mead is a traditional alcoholic beverage that results from the fermentation of diluted honey in water performed by yeasts. Sound scientific improvements have been achieved in several aspects of mead production: Honey-must formulation, fermentation and storage conditions and yeast performance [6,12,13,14,15,16,17,18,19,20,21]. Moreover, more than two dozen volatile compounds belonging to different chemical classes were identified and quantified in mead produced under different fermentation conditions [13,16,17,22,23]. However, only two studies have been focused on the mead sensory profile; one was about the influence of pollen addition on sensorial characteristics [18], while the other was specifically related to the sensory characteristics of mead produced with cassava floral honey [24]. Indeed, it is necessary to compare by way of sensory analysis, different meads, in order to assess the aroma and flavour characteristics of this type of beverage and for a better understanding of how its volatile profile may interfere with consumer acceptance.

Therefore, the first aim of this work was to characterize mead quality, through the identification and quantification of volatile compounds, produced by free and immobilized cells of two strains of *Saccharomyces cerevisiae*; QA23 and ICV D47. The second objective was to analyze the sensory properties of mead and to relate identified volatile compounds with aroma attributes.

## 2. Materials and Methods

### 2.1. Yeast Strains and Honey

*Saccharomyces cerevisiae* Lalvin QA23 (Lallemand, Montreal, QC, Canada) and Lalvin ICV D47 (Lallemand, Montreal, Canada) were used in this study as active wine dry yeasts.

For mead production, it was acquired from a local beekeeper of the northeast region of Portugal; a dark multifloral honey derived primarily from the pollen of *Castanea* spp. and *Erica* spp. The characteristics and satisfactory quality of the honey were assured in accordance with the requirements established in Portuguese law (Decreto-Lei n. 214/2003, 18 September, 2003).

### 2.2. Immobilization of Yeast Cells

Starter cultures were prepared by the rehydration of the active dry yeasts according to the manufacturer’s instructions, to obtain a cellular concentration, as colony forming units (CFU), of 10^8^ mL^−1^. To inoculate the honey-must, with a cellular concentration of 10^6^ mL^−1^, the appropriate amount of yeast suspension was added to a 40 g·L^−1^ sterilised sodium alginate (BDH Prolabo, Leuven, Belgium) solution. The polymer–cell mixture was added dropwise to a 180 mmol·L^−1^ CaCl_2_ (Panreac, Barcelona, Spain) sterilised solution and left to harden in this solution for 30 min at 4 °C. Beads containing immobilized *Saccharomyces cerevisiae* cells were rinsed three times with sterile distilled water. Then, the immobilized beads were transferred into the honey-must.

### 2.3. Honey-Must and Fermentation Conditions

The honey-must for fermentation with free or immobilized cells was prepared as described by Pereira et al. (2013) [16]. The honey was diluted in natural spring water (370 g·L^−1^) to obtain an alcoholic beverage with approximately 11% ethanol. Titratable acidity, as tartaric acid (TA), was adjusted to 5 g·L^−1^ with potassium tartrate (Sigma-Aldrich, St. Louis, MO, USA), and pH was adjusted to 3.7 with malic acid (Merck, Darmstadt, Germany). The nitrogen content, as yeast assimilable nitrogen (YAN), was adjusted to 267 mg·L^−1^ with diammonium phosphate (DAP, BDH Prolabo, Leuven, Belgium). The parameters pH, titratable acidity and YAN were determined after all the adjustments. The honey-must was divided into 2 L glass vessels and inoculated to obtain a cellular concentration, as *CFU*, of *S. cerevisiae* strain QA23 or ICV D47 in the immobilized or free form, of about 10^6^ mL^−1^. All fermentations were conducted in duplicate. The glass vessels were maintained during alcoholic fermentation at 25 °C under permanent but moderate shaking (120 min^−1^). Fermentations were monitored daily by weight loss, as an estimate of CO_2_ production and by quantification of the reducing sugars (*C*_RS_), using the 3,5-dinitrosalicylic acid (DNS) method with glucose as standard. At the end of alcoholic fermentation, with a duration of 7 days, the mead was centrifuged for further analyses (oenological, HPLC, volatile compounds and sensory determinations).

### 2.4. General Oenological Parameters

The oenological parameters, such as total sulphur dioxide (C_SO_2__), pH, titratable acidity, as tartaric acid (TA), volatile acidity, expressed as acetic acid (VA), and alcoholic strength by volume (ASv), were determined according to standard methods [25]. YAN was determined by the formaldehyde method as previously described [26].

### 2.5. HPLC Determination of Glucose, Fructose, Glycerol, Acetic Acid and Ethanol

Glucose, fructose, ethanol, glycerol and acetic acid were individually analysed, using a Varian high performance liquid chromatography (HPLC) system, equipped with a Rheodyne injector with a 20 µL loop, a Supelco Gel C—610 H column (300 mm × 7.8 mm) maintained at 35 °C and a refractive index detector RI-4 (Varian, Palo Alto, CA, USA). Isocratic elution was employed with a mobile phase consisting of 0.1% (v/v) phosphoric acid (Panreac, Barcelona, Spain) at a flow rate of 0.5 mL·min^−1^. Data were recorded and integrated using Star Chromatography Workstation software (Varian). Glucose, fructose, ethanol, glycerol and acetic acid were quantified by external standard calibration. 

### 2.6. Analysis of Mead Volatile Compounds

Mead produced with immobilized and free cells was analysed for major volatile compounds by Gas Chromatography with Flame-Ionization Detection (GC-FID) and for minor volatile compounds by Gas Chromatography Mass Spectrometry (GC-MS).

#### 2.6.1. Chromatographic Analysis of Major Volatile Compounds

In a glass tube, 100 µL of an ethanolic solution with 3540 mg·L^−1^ of internal standard (4-nonanol, Merck, Darmstadt, Germany) was added to 5 mL of mead.

A Chrompack GC CP-9000 gas chromatograph equipped with a split/splitless injector, a flame ionisation detector (FID) and a capillary column CP-Wax 57 CB (50 m × 0.25 mm; 0.2 μm film thickness) was used. The temperatures of the injector and detector were both set to 250 °C, and the split flow was 15 mL min^−1^. The column temperature was initially held at 60 °C for 5 min, then programmed to rise from 60 °C to 220 °C at 3 °C·min^−1^ and finally maintained at 220 °C for 10 min. The carrier gas was special helium 4× (Praxair, Danbury, CT, USA) at a flow rate of 1 mL·min^−1^ (125 kPa at the head of the column). The analysis was performed by the injection of 1 μL of sample. The major compounds in the samples were determined directly by the internal standard (4-nonanol) method, taking into account the relative response of the detector for each analyte, with Star–Chromatography Workstation software, version 6.41 (Varian). The identification was performed by comparing test compound retention times with those of pure standard compounds.

#### 2.6.2. Chromatographic Analysis of Minor Volatile Compounds

The extraction of mead minor volatiles was performed according to the method previously described [27]. In a 10 mL culture tube (Pyrex, ref. 1636/26MP), 8 mL of mead clarified by centrifugation, 100 µL of an ethanolic solution, 35.4 mg·L^−1^ of an internal standard (4-nonanol, Merck, Darmstadt, Germany) and a magnetic stir bar (22.2 mm × 4.8 mm) were added. The tube was sealed after addition of 400 µL of dichlorometane (Merck, Darmstadt, Germany) and extraction was accomplished by stirring the mixture for 15 min with a magnetic stirrer. After cooling the solutions at 0 °C for 10 min, the magnetic stir bar was removed, and the organic phase was separated by centrifugation (Relative Centrifugal Force [RCF] = 5118 for 5 min at 4 °C) and transferred into a vial with a Pasteur pipette. Finally, the aromatic extract was dried with anhydrous sodium sulphate (Merck, Darmstadt, Germany) and again transferred into a new vial.

Minor volatile compounds were analysed by GC-MS using a gas chromatograph Varian 3800 with a 1079 injector and an ion-trap mass spectrometer Varian Saturn 2000. A 1 µL injection was made in splitless mode (30 s) in a Varian Factor Four VF-WAXms (30 m × 0.15 mm; 0.15 µm film thickness) column. The carrier gas was helium UltraPlus 5× (99.9999%; Praxair, Danbury, CT, USA) at a constant flow rate of 1.3 mL·min^−1^ The detector was set to electronic impact mode with an ionisation energy of 70 eV, a mass acquisition. (*m*/*z*) from 35 to 260 and an acquisition interval of 610 ms. The oven temperature was initially 60 °C for 2 min and then raised from 60 °C to 234 °C at a rate of 3 °C·min^−1^, raised from 234 °C to 250 °C at 10 °C·min^−1^ and finally maintained at 250 °C for 10 min. The temperature of the injector was maintained at 250 °C during the analysis time, and the split flow was maintained at 30 mL min^−1^. The identification of compounds was performed using MS WorkStation version 6.6 software (Varian) by comparing their mass spectra and retention indices with those of pure standard compounds. The minor compounds were quantified in terms of 4-nonanol equivalents only.

### 2.7. Odour Activity Values

The odour activity values (OAV) is a measure of importance of a specific compound to the odour of a sample. The OAVs were calculated for each quantified volatile compound dividing the concentration of the compound by its perception threshold found in the literature [28,29,30,31,32].

### 2.8. Sensory Analysis

The evaluation of mead by sensory analysis was performed using the methodology described in Standards ISO 4121 (International Organisation for Standardization, 2003) and ISO 6658 (International Organisation for Standardization, 2005). The sensory attributes evaluated were divided into 3 groups: Appearance (colour and turbidity), taste (sweet, acid and astringency) and aroma (fruity, honey, vegetal, alcohol and chemical). These attributes were selected by reference to those normally used in sensory analysis of semi-sweet white wines. The intensity of each parameter was measured using a 7-point scale, corresponding 1 to “missing or invalid” and 7 “very strong”. Finally, the overall impression of each mead sample was evaluated using a scale of 1 to 10. All analyses were carried out by a panel of 16 semi-trained tasters.

### 2.9. Statistical Analysis

The chemical, HPLC and volatile data were analysed using a SPSS software, version 17.0 (SPSS, Inc., Chicago, IL, USA). To test significant differences among physicochemical characteristics and aromatic compounds of mead, a two factor—strain (S) and condition (C)—analysis of variance (ANOVA) was applied. The fulfilment of the ANOVA requirements, namely the normal distribution of the residuals and the homogeneity of variance, was evaluated by means of the Shapiro–Wilks test (*n* < 50) and Levene’s test, respectively. All statistical tests were performed at a 5% significance level.

The sensory data was analysed using the software XLstat program with Excel from Microsoft Office, following the internet tutorial from XLSTAT (PrefMap) (2006).

Principal component analysis (PCA) is one of the most commonly used multivariate techniques for grape and wine analysis [11,33]. PCA was used to relate the concentration of volatile compounds with OAV > 1 and the aroma attributes with different meads. PCA was applied to pre-process and reduce the dimensionality of the multivariate chemical and sensorial data using the statistics program R, version 3.2.0 (The R Foundation for Statistical Computing, Vienna, Austria), a free software environment for statistical computing and graphics. The R statistical packages FactoMineR [33] and factoextra [34] were used.

## 3. Results and Discussion

### 3.1. General Physicochemical Characterization of Mead

The values of the classical physicochemical parameters of mead produced by the strains QA23 and ICV D47, under free or immobilized form, are displayed in Table 1.

The pH and volatile acidity were significantly influenced by the factor strain (S) (QA23 or ICV D47) and yeast condition (C) (free or immobilized cells). Both strains increased pH values and volatile acidity in fermentations with immobilized cells compared to those performed by free cells. The pH of mead varied from 3.46 to 3.53, being lower for strain ICV D47. Volatile acidity, in mead fermented by the strain QA23 in free and immobilized form was 0.57 g·L^−1^ and 0.69 g·L^−1^, respectively. These values were significantly higher than those obtained in mead produced by the strain ICV D47, irrespective of the yeast condition (0.51 g·L^−1^ and 0.54 g·L^−1^). Slightly lower values of volatile acidity have been reported in mead obtained with free cells [16]. The use of high fermentations volumes in this work probably has modified the fermentation conditions, which affected yeast growth, and therefore, modulate the accumulation of acetic acid [35]. Total SO_2_ was significantly lower in mead fermented with immobilized cells. In sum, the effect of strain on the physicochemical characteristics of mead was not affected by the yeast-condition as indicated by non-significant interaction between the two factors for all parameters analysed.

The concentrations of sugars, glucose and fructose, and fermentation products, ethanol, glycerol and acetic acid, at the end of fermentations, are shown in Table 2.

The final concentrations of fructose, glycerol and acetic acid in mead were dependent on the yeast strain. The strain ICV D47 has consumed less fructose than the strain QA23, resulting in a mead with higher residual fructose (3.67 g·L^−1^ and 3.05 g·L^−1^ for free and immobilized cells, respectively). On the other hand, the strain QA23 produced higher amounts of glycerol and acetic acid, either in free or immobilized form. Further, the consumption of fructose and production of acetic acid were also dependent on the yeast cell condition: Free cells consumed less fructose and conversely, immobilized cells produced more acetic acid. Accordingly, the interaction between the strain and yeast condition was statistically significant, in respect to fructose concentration. The values of acetic acid ranged from 0.21 g·L^−1^ to 0.39 g·L^−1^, which were lower than the volatile acidity. In fact, volatile acidity includes a group of volatile organic acids, such as acetic acid, which comprises about 90% of volatile acids, propionic and hexanoic acids, among others [4].

### 3.2. Mead Volatile Compounds

Alcoholic fermentation results not only in ethanol and carbon dioxide production by yeast but also in a complex mixture of flavour-active by-products. Eight major volatile compounds, including acetaldehyde, ethyl acetate, methanol, 1-propanol, 2-methyl-1-propanol, 2-methyl-1-butanol, 3-methyl-1-butanol and 2-phenylethanol were identified by GC-FID. The minor compounds quantified by GC-MS were ethyl butyrate, isoamyl acetate, ethyl hexanoate, ethyl lactate, 3-ethoxy-1-propanol, ethyl octanoate, isobutyric acid, butanoic acid, ethyl decanoate, 3-(methylthio)-1-propanol, ethyl phenylacetate, 2-phenylethyl acetate, ethyl dodecanoate, hexanoic acid, octanoic acid, 4-vinylguaicol, decanoic acid, 4-vinylphenol and dodecanoic acid. The concentrations of volatile compounds in mead produced by the two strains of *S.cerevisiae*, QA23 and ICV D47, either in immobilized or free form are shown in Table 3. In total, twenty-seven compounds were quantified, including alcohols, esters, volatile phenols, volatile fatty acids and carbonyl compounds.

The alcohols were quantitatively the largest group of volatile compounds and 3-methyl-1-butanol was the major compound in all meads. Alcohols are, from a quantitative point of view, the major group of volatile compounds produced by yeast during alcoholic fermentation [35]. Concentrations of higher alcohols below 300 mg·L^−1^ add a desirable level of complexity to wine, whereas concentrations that exceed 400 mg·L^−1^ can have a detrimental effect [4]. The yeast strain used had a significant effect on the production of methanol and 3-ethoxy-1-propanol, instead the condition influenced the production of five alcohol compounds (methanol, 2-methyl-1-propanol, 2-methyl-1-butanol, 3-methyl-1-butanol, and 3-(methylthio)-1-propanol). Even so, four alcohol compounds, (2-methyl-1-propanol, 2-methyl-1-butanol, 3-methyl-1-butanol and 3-(methylthio)-1-propanol), presented a significant interaction between the factors strain and condition. The alcohol 3-ethoxy-1-propanol was produced in significantly lower amounts in fermentations conducted with strain ICV D47, regardless of the yeast condition. Similar results have already been obtained in mead produced with this strain under other fermentation conditions [16,17]. In general, independently of the strain, the immobilization of yeast cells led to lower concentrations of 2-methyl-1-propanol, 2-methyl-1-butanol and 3-methyl-1-butanol.

The esters were the second largest group of quantified volatile compounds. The production of esters by the yeasts during fermentation can have a significant effect on the fruity flavours in alcoholic beverages [4,36]. Comparatively with alcohols, a smaller number of esters showed significant differences among strains or conditions. The strain QA23 in free form did not produce ethyl decanoate, whereas the strain ICV D47 produced it in higher concentration in free than in immobilized form, leading to a significant interaction S × C. A significant effect of the yeast-condition was observed in the production of ethyl acetate, ethyl phenylacetate and ethyl dodecanoate. Ethyl acetate was the major ester found in mead, which had a concentration that varied from 28.02 mg·L^−1^ to 50.07 mg·L^−1^ in mead produced with immobilized cells. Similar results have already been reported in mead [17] or in white wine [37,38] produced with free and immobilized cells. The reverse was observed for ethyl phenyl-acetate and ethyl dodecanoate, i.e., significantly lower concentrations were found in mead obtained by immobilized cells.

Volatile phenols are formed by decarboxylation of hydroxycinnamic acid precursors, *p*-coumaric, caffeic and ferulic acids [39]. These acids have also been detected in chestnut, sunflower, lavender and acacia honeys [40]. Vinylphenols, particularly 4-vinylguaiacol and 4-vinylphenol, are responsible for producing a pharmaceutical odour [4]. The volatile phenol, 4-vinylphenol, presented the highest concentration in mead, however only the production of 4-vinylguaicol was significantly influenced by the yeast cell condition. This compound was detected in higher concentrations in fermentations with free cells. Similar results have been recently reported in wine assays using immobilized cells [26,38].

Volatile fatty acids are produced through the lipid metabolism by yeast and are usually associated with unpleasant aromas, such as fatty, sweat, rancid or cheese [30]. Six fatty acids were identified and quantified in all fermentations, being octanoic acid the main compound, as already reported in a previous work with the same type of beverage [16]. The concentration of dodecanoic acid showed significant differences among yeast cell conditions, being higher in fermentations performed by immobilized cells.

Acetaldehyde is the major carbonyl compound found in wine, contributing to flavour with aroma descriptors such as ‘bruised apple’ and ‘nutty’ but can also be associated with oxidation off-flavours at high concentrations [4,35]. The concentration of acetaldehyde was dependent on yeast cell condition; higher amounts were detected in mead produced with free cells, approximately 13 mg·L^−1^ and 16 mg·L^−1^ for strain ICV D47 and QA23, respectively. These results are in agreement with the ones reported in wine by Genisheva (2013) [38] and Genisheva et al. (2014) [27], who also noted higher amounts of acetaldehyde in wines produced with free cells. Nevertheless, probably due to different fermentation conditions, the values found in this work are higher than those previously reported in mead [16,17,18].

### 3.3. Odour Activity Values

The odour activity values (OAVs) were determined in order to evaluate the potential contribution of each volatile compound to the mead aroma. Only the compounds with an OAV > 1 are potential contributors to the beverage aroma [31]. Taking into account the great variability of the sensory thresholds found in literature, the authors believe that the computation of the exact value of volatile compounds is less important. Thereby, in this study, semi-quantitative results we obtained for the volatile compounds which allowed us to identify the most relevant compounds capable of being correlated with sensory descriptive data.

Thus, from the twenty-seven volatile compounds quantified, only fifteen were above their perception threshold (Table 4).

The alcohols, 3-methyl-1-butanol and 2-phenylethanol were present above its odour threshold, particularly in mead produced with free cells. The alcohol, 2-phenylethanol, is generally a positive contributor to fermented beverages aroma, being characterized by a pleasant rose-like aromatic alcohol [4].

Among the esters, a total of seven compounds presented OAV > 1, being isoamyl acetate, ethyl hexanoate and ethyl octanoate the most aromatic. The three esters may have contributed to the beverage with fruity/floral characteristics [31,32], although no significant differences were observed in their concentrations among strains or cell conditions (Table 3).

The volatile phenols, 4-vinylphenol and 4-vinylguaicol, were only detected in concentrations above its perception threshold (OAV > 1) in mead produced with free cells. Normally, volatile phenols have a low influence in the aroma of most wines, but concentrations above certain limits can have a negative effect and depreciate the aroma of wine by masking the fruity character and giving phenolic off-flavours [41].

Three volatile fatty acids, usually associated with unpleasant aromas of fatty, rancid and cheese, were detected in all mead above their odour perception threshold, being octanoic acid present in higher concentrations in mead produced with free cells. Although, according to ANOVA, no statistically significant differences were observed between conditions (Table 3). These volatile compounds are precursors of esters, like ethyl octanoate, associated with a fruity and sweet aroma, which exhibited the highest OAV in mead produced by the strain QA23 in free form (OAV = 183.5).

Acetaldehyde was one of the most aromatic compounds, and its contribution was particularly relevant in mead produced with free cells, being three to four times higher compared to fermentations with immobilized cells.

In general, the OAVs revealed that the mead produced using free cells presented a more interesting aroma profile. The opposite was observed in a previous work about mead produced in smaller volumes with the same strains in free and immobilized forms [17].

**Figure 1:**
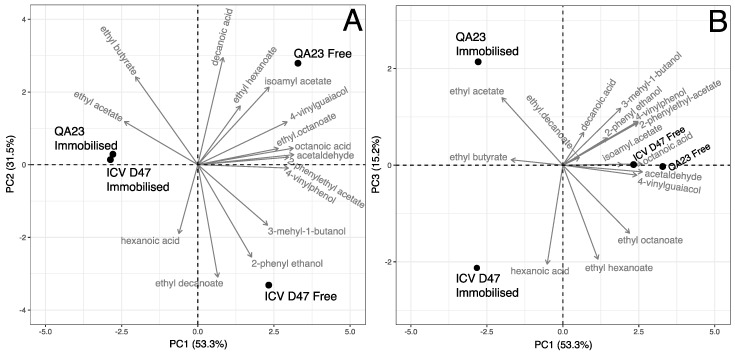
Principal component analysis (PCA) plots, using the concentrations of volatile compounds quantified in meads obtained using the two strains, QA23 and ICV D47, in free or immobilized forms. PCA biplots of volatile compounds data present in meads obtained by the strains QA23 and ICV D47, with free or immobilized cells: (**A**) PC1 vs. PC2; (**B**) PC1 vs. PC3.

To obtain a more simplified view of the relationship between the different meads produced and their volatile composition, a principal component analysis (PCA) was performed using the aroma compounds with OAVs > 1 (Figure 1).

The above is an unsupervised statistical method used for data exploration and visualization, which allows data multivariate pattern recognition and dimensionality reduction without prior knowledge about the samples. The results showed that 100% of the data variability of the volatile compounds was explained with three principal components, PCs (using centred and scaled data). The first two principal components, PC1 and PC2, accounted for 53.3% and 31.5% of the total variance, respectively (using centred and scaled data). Figure 1 shows two graphical representations of the bi-dimensional spaces defined by PC1 vs. PC2 and PC1 vs. PC3.

This approach allowed the identification of the volatile compounds that best discriminated the meads. The first component, PC1, was characterized by higher levels of isoamyl acetate, 2-phenylethyl acetate, 4-vinylguaicol, 4-vinylphenol, octanoic acid, ethyl hexanoate, ethyl octanoate and acetaldehyde. Concerning the second principal component, PC2, the volatile compounds 2-phenylethanol and 3-methyl-1-butanol showed the highest and positive values, while ethyl acetate and ethyl butyrate contributed to the negative side of the same principal component. PC1 discriminated mead produced by strain QA23 with free or immobilized cells, while PC2 discriminated mead produced by strain ICV D47.

The PC1, in the positive zone, was mainly characterized by high concentrations of the volatile compounds 3-mehyl-1-butanol, isoamyl acetate, ethyl octanoate, 4-vinylguaiacol, 4-vinylphenol, 2-phenylethyl acetate, octanoic acid and acetaldehyde. These volatile compounds were ordered by increasing contributions to the result of the principal component, ranging between 6.5% and 12.4%. Ethyl butyrate and ethyl acetate were the only variables to contribute with high values to the negative zone, having contributions of 5.2% and 7.2%, respectively, for the PC1 overall result. Figure 1A shows that PC1 allowed us to separate the mead produced with free yeast cells that appeared in the PC1 positive values zone from those produced with immobilized yeast cells that appeared in the PC1 negative values zone. This means that the latter samples had high concentration values in volatile compounds ethyl butyrate and ethyl acetate and lower concentrations of the volatile compounds 3-mehyl-1-butanol, isoamyl acetate, ethyl octanoate, 4-vinylguaiacol, 4-vinylphenol, 2-phenylethyl acetate, octanoic acid and acetaldehyde. The mead produced with free yeast cells show reverse behavior as they are in the positive zone of PC1.

For PC2, the highest values of the volatile compounds ethyl hexanoate, isoamyl acetate, ethyl butyrate and decanoic acid contributed to the principal component positive values, being presented in ascending order of influence to the result of this principal component (contributions between 5.5% and 18.3%); while, 3-mehyl-1-butanol, hexanoic acid, 2-phenyl ethanol and ethyl decanoate were the main contributors (sorted by increasing percentage contribution values, ranging between 5.8% to 20.2%) to the high values in the negative zone of PC2. In Figure 1A, PC2 allowed to differentiate the mead produced by the two strains with free cells process, being the mead produced with the QA23 strain free cells in the positive zone meaning due to high concentration values in volatile compounds of ethyl hexanoate, isoamyl acetate, ethyl butyrate and decanoic acid, as well low concentration values in volatile compounds of 3-mehyl-1-butanol, hexanoic acid, 2-phenyl ethanol and ethyl decanoate. The mead from immobilized ICV D47 cells had the reverse pattern since it appeared in the PC2 negative zone.

In the dimension PC3, high negative values were mainly associated to volatile compounds ethyl octanoate, ethyl hexanoate and hexanoic acid, in ascending order, which present contributions to the PC between 12.5% and 26.6%. Regarding the high positive values, the contributions are especially the 4-vinylphenol, 3-mehyl-1-butanol and ethyl acetate compounds (representing 5.2% to 12.4% of the overall PC result). As it can be seen in Figure 1B, the mead samples obtained with immobilized cells were separated in the PC3 dimension, with the QA23 strain and CVD47 strain, in the positive and negative zones, respectively. So, mead from immobilized ICV D47 cells presented higher concentrations of ethyl octanoate, ethyl hexanoate and hexanoic acid compounds as well as lower concentrations of 4-vinylphenol, 3-mehyl-1-butanol and ethyl acetate compounds. The reverse pattern was obtained for the mead from immobilized QA23 cells.

In general, meads produced with free cells were characterized by the presence of volatile compounds associated with pleasant aromas. Particularly, mead produced using the strain QA23 was characterized by the global influence of volatile compounds with odour descriptors of fruity, sweet, aniseed, banana, fatty and soap; while the mead of strain ICV D47 presented volatile compounds with odour descriptors of cheese, nail polish, roses, flowery, pleasant and soap. Instead, mead produced with immobilized cells were differentiated due to the presence of volatile compounds with less pleasant odour descriptors of as solvent, nail polish, fruity and sweet.

### 3.4. Mead Sensory Analysis

Mead samples were subjected to a sensory characterization in order to evaluate the effect of strain and their form (free or immobilized) in the aroma and flavour. The analysis was performed by a panel of 16 semi-trained tasters using a total of 10 sensory attributes: Two for appearance (colour and turbidity), three for taste (sweet, acid and astringency) and five for aroma (fruity, honey, vegetal, alcohol and chemical).

**Figure 2:**
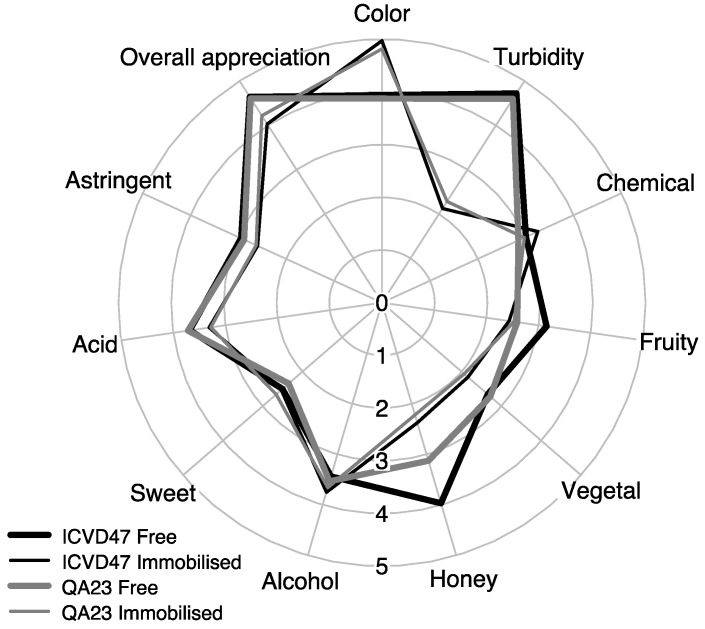
Radar plot of the mean aroma descriptors obtained for the meads produced using the two strains, QA23 and ICV D47, with free or immobilized cells.

Figure 2 shows a radar plot that depicts the results of the aroma descriptors obtained for the four mead samples under study. The radar plot shows that the mead obtained by the two strains, QA23 and ICV D47, with free cells had similar sensory profile, which was not the case for the mead produced with immobilized cells, which presented greater differences mainly in the honey and fruity descriptors.

For interpreting the results, PCA was applied to identify the aroma descriptors that better discriminated mead obtained by the two strains mentioned above (Figure 2).

The first two principal components accounted for 92.13% of total variance. PC1, which accounted for 79.62% of total variance, clearly discriminated mead produced with free or immobilized cells. The first component, PC1, was highly positively correlated with turbidity and astringency and so, the appearance and taste were the greatest contributors to discriminating mead produced by free yeast cells, which was also correlated to the following sensory aroma attributes: acid, vegetal and honey. Meads produced with immobilized cells located at the negative side of the PC1 and were correlated to the attributes of colour and alcohol. PC2 accounted for 12.51% of variance and the attributes of alcohol and vegetal showed high and positive values and sweet and fruity contributed to the negative side of same PC.

**Figure 3:**
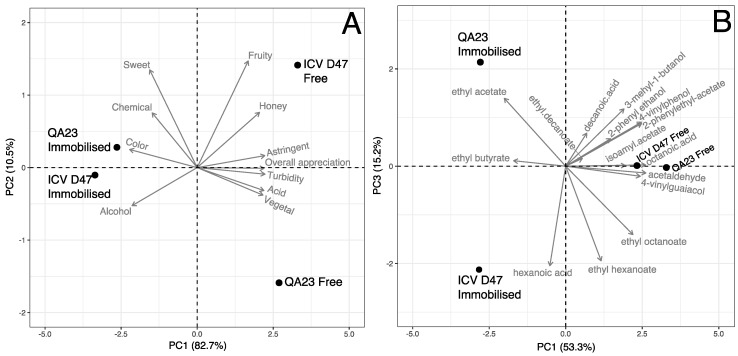
Principal component analysis (PCA) plot of mead obtained by the two strains, QA23 and ICV D47, with free or immobilized cells. PCA biplots of volatile compounds data present in meads obtained by the strains QA23 and ICV D47, with free or immobilized cells: (**A**) PC1 vs. PC2; (**B**) PC1 vs. PC3.

PCA was also performed in order to analyse the natural variability within the aroma descriptors data of the four mead samples. The three PCs explained 100% of data variance: PC1 explains 82.7%; PC2 describes 10.5%; and PC3, 6.8%. Figure 3 presents two PCA biplots that correspond to the bi-dimensional spaces defined by PC1 vs. PC2 and PC1 vs. PC3.

The dimension PC1 had the contribution of all aroma descriptors (representing contributions > 4.8% to the principal component) being the most relevant variables vegetal, acid, overall appreciation, astringent and turbidity for higher positive values (contributions > 10.3%), as well the variables alcohol and colour for higher negative values (contributions > 10.1%). Figure 3A shows that in the PC1 axis, mead samples prepared with free cells are present in the positive zone and separated from those obtained with immobilized cells, which are present in the negative zone. So, mead from free yeast cells were characterized with high values in vegetal, acid, overall appreciation, astringent and turbidity descriptors as well, low values in alcohol and colour descriptors. The reverse situation was obtained for the mead produced from immobilized yeast cells.

The PC2 was represented mainly by the variables that contribute to the high positive values as chemical, honey, sweet and fruity (contributions between 9.9% and 37.6%). Figure 3A allows to verify that mead obtained with free ICV D47 cells had higher classification values in those variables, contrary to the mead produced with free QA23 cells, which had low grades in the descriptors associated with PC2.

The PC3 dimension highlights the main contribution of one aromatic descriptor to high positive values, the chemical descriptor (contribution of 60.0% to the PC); and another descriptor to the large negative values, sweet (contribution of 19.8% to the PC). Figure 3B allows to verify that in the PC3 dimension, samples produced with immobilized cells were also separated. The mead produced with immobilized ICV D47 cells had high and low classifications in the chemical and sweet descriptors, which corresponds to the opposite situation of the mead sample obtained with immobilized QA23 cells.

The sensory analysis results reflect the degree of clarification as a factor of distinction between the mead produced by immobilized and free cells. In fact, the results obtained in sensory analysis reflect the degree of clarification of mead produced by immobilized cells, correlated with appearance (colour), whereas meads obtained with free cells were correlated with the attribute turbidity. The strain and the yeast-condition had a significant effect on the volatile acidity and therefore on the acetic acid concentration, which were higher in mead produced by the strain QA23 in the immobilized form (Table 1 and Table 2). However, the sensory analysis showed that the acid attribute was more perceptible in mead obtained with free cells process. In general, the overall appreciation revealed that tasters preferred mead produced with free cells. Considering the results obtained in the PCA of the 15 volatile compounds with OAVs > 1 and in the PCA with the sensory descriptors, correlations can be tentatively established since Figure 1 and Figure 3 show some similarity in the spatial distribution of mead samples. Therefore, as previously referred, the sensory analysis indicated that taste and aroma descriptors which better discriminate mead produced by yeast free cells were acid, astringency, vegetal, overall appreciation and turbidity (Figure 3) that are possibly related to the volatile compounds that better characterized that type of mead, namely 3-mehyl-1-butanol, isoamyl acetate, ethyl octanoate, 4-vinylguaiacol, 4-vinylphenol, 2-phenylethyl acetate, octanoic acid and acetaldehyde (Figure 1). Although the results suggest that mead produced by free cells of the strain QA23 was more aromatic (compounds with higher OAVs) than that produced using the strain ICV D47 (Table 4 and Figure 1), such difference was not perceptible by the taster panel. Unlike mead produced with free cells of the strain ICV D47, those obtained with immobilized cells were characterized by volatile compounds associated with unpleasant aromas, such as ethyl butyrate and ethyl acetate (Figure 1), which that could be noticeable in sensory analysis as the chemical aroma attribute (Figure 3).

## 4. Conclusions

This study is one of the first approaches combining volatile composition and sensory properties of mead. Two strains, QA23 and ICV D47, in free or immobilized form were used to produce four different meads. The strain and yeast cell conditions had a significant effect on some characteristics of the final product, such as pH, volatile acidity, fructose and volatile compounds’ concentrations. Only fifteen volatile compounds out of the twenty-seven quantified, were above their perception threshold, and therefore were potential contributors to mead aroma. This work reveals a relationship between the volatile characteristics and sensory properties of mead. The sensory analysis allowed to distinguish mead produced with free and immobilized cells. Regarding strains, the ICV D47 behaved differently in terms of aroma compounds formation in free or immobilized cells, but these differences were less pronounced for the strain QA23. In general, yeast cell conditions (free or immobilized) had more influence than the strain on the sensory characteristics of final product. Despite some off-flavour compounds detected in mead produced with free cells, they were overall more appreciated by the taste panel.

Considering the results obtained in respect to sensory properties of mead and to have a better understanding on the correlation between volatile composition and sensory properties, further studies focused on sensory quality should be performed.

## Figures and Tables

**Table 1:** Physicochemical characteristics (mean values and standard deviation) of mead fermented by *S. cerevisiae* QA23 and ICV D47 with free cells (F) or immobilized cells (I) and significance of the factors strain (S) and condition (C) according to two-way ANOVA.

Parameters	QA23–F	QA 23–I	ICV D47–F	ICV D47–I	Significance		
					Strain	Condition	S × C
pH	3.48 ± 0.01	3.53 ± 0.01	3.46 ± 0.02	3.47 ± 0.01	0.021	0.047	ns
*VA* (g·L^−1^)	0.57 ± 0.04	0.69 ± 0.04	0.51 ± 0.04	0.54 ± 0.00	0.016	0.045	ns
*TA* (g·L^−1^)	5.79 ± 0.03	5.38 ± 0.19	5.87 ± 0.29	5.53 ± 0.29	ns	ns	ns
*YAN* (mg·L^−1^)	31.50 ± 4.95	29.75 ± 2.47	33.25 ± 2.47	36.75 ± 2.47	ns	ns	ns
*C*_SO_2__ (mg·L^−1^)	29.44 ± 1.81	24.32 ± 1.81	26.88 ± 1.81	25.60 ± 0.00	ns	0.045	ns
*ASv* (%)	11.38 ± 0.18	11.13 ± 0.18	11.13 ± 0.18	11.00 ± 0.35	ns	ns	ns
*C*_RS_ (g·L^−1^)	24.31 ± 5.88	24.66 ± 0.98	25.70 ± 3.43	21.71 ± 0.49	ns	ns	ns

C_SO_2__—Mass concentration of SO_2_; ASv—alcoholic strength by volume; TA—titratable acidity, expressed as tartaric acid; VA—volatile acidity, expressed as acetic acid; YAN—yeast assimilable nitrogen; C_RS_—reducing sugars concentration, expressed as glucose. ns—indicates no significant difference (*p* > 0.05).

**Table 2:** Concentration of sugars, glycerol, acetic acid and ethanol (mean values and standard deviation) of mead fermented by *S. cerevisiae* QA23 and ICV D47 with free cells (F) or immobilized cells (I) and significance of the factors strain (S) and condition (C) according to two-way ANOVA.

Parameters	QA23–F	QA23–I	ICV D47–F	ICV D47–I	Significance		
					Strain	Condition	S × C
Glucose (g·L^−1^)	1.78 ± 0.53	1.72 ± 0.03	1.84 ± 0.23	1.69 ± 0.07	ns	ns	ns
Fructose (g·L^−1^)	2.72 ± 0.06	2.66 ± 0.16	3.67 ± 0.14	3.05 ± 0.14	0.002	0.021	0.040
Glycerol (g·L^−1^)	5.23 ± 0.19	5.14 ± 0.08	5.07 ± 0.21	4.43 ± 0.25	0.032	ns	ns
Acetic acid (g·L^−1^)	0.30 ± 0.02	0.39 ± 0.03	0.21 ± 0.01	0.29 ± 0.01	0.001	0.002	ns
Ethanol (%)	9.63 ± 0.05	10.12 ± 0.06	10.36 ± 0.15	9.54 ± 0.78	ns	ns	ns

ns—indicates no significant difference (*p* > 0.05).

**Table 3:** Concentration of volatile compounds (mean values and standard deviation) of mead fermented by *S. cerevisiae* QA23 and ICV D47 with free cells (F) or immobilized cells (I) and significance of the factors strain (S) and condition (C) according to two-way ANOVA.

Compounds	QA23–F	QA23–I	ICV D47–F	ICV D47–I	Significance		
					Strain	Condition	S × C
**Alcohols (mg L^−1^)**							
methanol	3.25 ± 0.66	4.82 ± 1.01	1.09 ± 0.24	4.01 ± 0.24	0.028	0.007	ns
1-propanol	32.28 ± 6.04	41.87 ± 1.14	40.15 ± 8.98	65.37 ± 18.23	ns	ns	ns
2-methyl-1-propanol	15.73 ± 2.04	14.74 ± 0.69	18.27 ± 1.05	10.77 ± 0.13	ns	0.007	0.018
2-methyl-1-butanol	13.36 ± 0.47	11.04 ± 1.79	16.61 ± 0.32	8.79 ± 0.35	ns	0.002	0.015
3-methyl-1-butanol	104.72 ± 3.49	99.65 ± 14.05	125.18 ± 9.75	78.79 ± 6.16	ns	0.017	0.034
3-ethoxy-1-propanol	0.18 ± 0.01	0.16 ± 0.01	0.02 ± 0.01	0.009 ± 0.004	0.000	ns	ns
3-(methylthio)-1-propanol	0.0136 ± 0.0003	0.010 ± 0.004	0.04 ± 0.01	0.005 ± 0.002	ns	0.008	0.018
2-phenylethanol	24.59 ± 5.19	24.78 ± 6.48	35.50 ± 3.29	21.64 ± 5.68	ns	ns	ns
**Total**	**194.12 ± 8.96**	**197.07 ± 15.67**	**236.85 ± 13.71**	**189.39 ±20.07**			
**Esters (mg L^−1^)**							
ethyl acetate	34.28 ± 0.81	50.07 ± 11.83	28.02 ± 1.61	38.03 ± 2.94	ns	0.041	ns
ethyl butyrate	0.15 ± 0.04	0.167 ± 0.003	0.08 ± 0.01	0.16 ± 0.06	ns	ns	ns
isoamyl acetate	1.49 ± 0.16	1.13 ± 0.33	1.16 ± 0.40	1.12 ± 0.27	ns	ns	ns
ethyl hexanoate	0.47 ± 0.05	0.31 ± 0.10	0.37 ± 0.13	0.44 ± 0.08	ns	ns	ns
ethyl lactate	0.07 ± 0.01	0.05 ± 0.01	0.08 ± 0.03	0.04 ± 0.01	ns	ns	ns
ethyl octanoate	0.92 ± 0.21	0.45 ± 0.01	0.80 ± 0.08	0.71 ± 0.23	ns	ns	ns
ethyl decanoate	nd	0.24 ± 0.02	0.82 ± 0.25	0.19 ± 0.01	0.013	ns	0.009
ethyl phenylacetate	0.017 ± 0.005	0.007 ± 0.001	0.013 ± 0.001	0.0033 ± 0.0003	ns	0.005	ns
2-phenylethyl acetate	0.74 ± 0.22	0.51 ± 0.09	0.67 ± 0.13	0.37 ± 0.06	ns	ns	ns
ethyl dodecanoate	0.08 ± 0.03	0.007 ± 0.002	0.12 ± 0.07	0.007 ± 0.004	ns	0.030	ns
**Total**	**38.21 ± 1.20**	**52.94 ± 11.83**	**32.14 ± 1.69**	**41.07 ± 2.97**			
**Volatile phenols (μg·L^−1^)**							
4-vinylguaiacol	128.11 ± 27.76	53.15 ± 3.72	87.76 ± 9.67	59.03 ± 4.80	ns	0.008	ns
4-vinylphenol	183.67 ± 28.65	157.34 ± 8.41	179.66 ± 5.62	139.85 ± 16.90	ns	ns	ns
**Total**	**311.78 ± 39.89**	**210.48 ± 9.20**	**267.42 ± 11.18**	**198.89 ± 17.57**			
**Volatile fatty acids (μg L^−1^)**							
isobutyric acid	25.80 ± 0.71	19.41 ± 0.43	40.12 ± 19.15	17.06 ± 4.90	ns	ns	ns
butanoic acid	20.89 ± 2.68	15.07 ± 3.03	29.13 ± 11.76	15.52 ± 5.44	ns	ns	ns
hexanoic acid	714.12 ± 95.56	713.94 ± 14.99	757.47 ± 98.22	769.58 ± 296.92	ns	ns	ns
octanoic acid	3224.03 ± 282.58	2825.68 ± 293.58	3094.58 ± 758.90	2817.21 ± 335.32	ns	ns	ns
decanoic acid	1263.80 ± 71.73	1178.30 ± 178.95	1081.01 ± 354.72	1126.96 ± 204.77	ns	ns	ns
dodecanoic acid	3.48 ± 1.80	10.69 ± 1.26	2.55 ± 0.90	8.72 ± 1.69	ns	0.003	ns
**Total**	**5252.12 ± 306.82**	**4763.09 ± 344.16**	**5004.86 ± 843.74**	**4755.06 ± 492.53**			
**Carbonyl compounds (mg L^−1^)**							
Acetaldehyde	15.80 ± 2.25	3.63 ± 0.28	12.72 ± 2.33	4.32 ± 0.53	ns	0.001	ns

ns—indicates no significant difference; nd—indicates not detected.

**Table 4:** Odor activity values (OAV) of volatile compounds of more influence on the aroma of mead fermented by *S. cerevisiae* QA23 and ICV D47 with free cells (F) or immobilized cells (I).

Compounds	Odour Descriptor ^a^	Odour Threshold (μg·L^−1^)	QA23-F	QA23-I	ICV D47-F	ICV D47-I
3-methyl-1-butanol	cheese; nail polish	30,000	3.5	3.3	4.2	2.6
2-phenyl-ethanol	roses; flowery	14,000	1.8	1.8	2.5	1.5
ethyl acetate	solvent; nail polish	12,300	2.8	4.1	2.3	3.1
ethyl butyrate	fruity; sweet	20	7.4	8.3	4.2	8.1
isoamyl acetate	banana	30	49.7	37.5	38.6	37.3
ethyl hexanoate	fruity; aniseed	14	33.6	22.4	26.7	31.3
ethyl octanoate	fruity; sweet	5	**183.5**	**89.9**	**159.7**	**141.3**
ethyl decanoate	pleasant; soap	200	nd	1.2	4.1	< 1
2-phenylethyl acetate	flowery; roses	250	3.0	2.0	2.7	1.5
4-vinylguaicol	clove	130	1.0	< 1	< 1	< 1
4-vinylphenol	almond shell	180	1.0	< 1	1.0	< 1
hexanoic acid	cheese; sweaty	420	1.7	1.7	1.8	1.8
octanoic acid	fatty; rancid	500	6.4	5.7	6.2	5.6
decanoic acid	fatty; soap	1000	1.3	1.2	1.1	1.1
Acetaldehyde	fresh; green	500	31.6	7.3	25.4	8.6

^a^ Odor descriptors reported in the literature and; nd—not detected.

## References

[1] Pascoal A., Anjos O., Feás X., Oliveira J.M., Estevinho L.M. (2018). Impact of fining agents on the volatile composition of sparkling mead. J. Inst. Brew..

[2] Mendes-Ferreira A., Barbosa C., Lage P., Mendes-Faia A. (2011). The impact of nitrogen on yeast fermentation and wine quality. Cienc. Tecn. Vitivinic..

[3] Robinson A.L., Boss P.K., Heymann H., Soloman P.S., Trengove R.D. (2011). Influence of yeast strain, canopy management, and site on the volatile composition and sensory attributes of Cabernet Sauvignon wines from Western Australia. J. Agric. Food Chem..

[4] Swiegers J.H., Bartowsky E.J., Henschke P.A., Pretorius I.S. (2005). Yeast and bacterial modulation of wine aroma and flavour. Aust. J. Grape Wine Res..

[5] Vilanova M., Oliveira J.M., Salih B., Çelikbiçak O. (2012). Application of Gas Chromatography on the Evaluation of Grape and Wine Aroma in Atlantic Viticulture (NW Iberian Peninsula). Gas Chromatography in Plant Science, Wine technology, Toxicology and Some Specific Applications.

[6] Chen C.-H., Wu Y.-L., Lo D., Wu M.-C. (2013). Physicochemical property changes during the fermentation of longan (Dimocarpus longan) mead and its aroma composition using multiple yeast inoculations. J. Inst. Brew..

[7] Gupta J.K., Sharma R. (2009). Production technology and quality characteristics of mead and fruit-honey wines: A review. Nat. Prod. Radiance.

[8] Andreu-Sevilla A.J., Mena P., Martí N., Viguera C.G., Carbonell-Barrachina A.A. (2013). Volatile composition and descriptive sensory analysis of pomegranate juice and wine. Food Res. Int..

[9] Schmidtke L.M., Rudnitskaya A., Saliba A.J., Blackman J.W., Scollary G.R., Clarck A.C., Rutledge D.N., Delgadillo I., Legin A. (2010). Sensory, chemical, and electronic tongue assessment of micro-oxygenated wines and oak chip maceration: Assessing the commonality of analytical techniques. J. Agric. Food Chem..

[10] Smyth H., Cozzolino D. (2013). Instrumental methods (spectroscopy, electronic nose, and tongue) as tools to predict taste and aroma in beverages: Advantages and limitations. Chem. Rev..

[11] Vilanova M., Genisheva Z., Masa A., Oliveira J.M. (2010). Correlation between volatile composition and sensory properties in Spanish Albariño wines. Microchem. J..

[12] Gomes T., Barradas C., Dias T., Verdial J., Morais J.S., Ramalhosa E., Estevinho L.M. (2013). Optimization of mead production using Response Surface Methodology. Food Chem. Toxicol..

[13] Mendes-Ferreira A., Cosme F., Barbosa C., Falco V., Inês A., Mendes-Faia A. (2010). Optimization of honey-must preparation and alcoholic fermentation by *Saccharomyces cerevisiae* for mead production. Int. J. Food Microbiol..

[14] Navrátil M., Šturdík E., Gemeiner P. (2001). Batch and continuous mead production with pectate immobilised, ethanol-tolerant yeast. Biotechnol. Lett..

[15] Pereira A.P., Dias T., Andrade J., Ramalhosa E., Estevinho L.M. (2009). Mead production: Selection and characterization assays of *Saccharomyces cerevisiae* strains. Food Chem. Toxicol..

[16] Pereira A.P., Mendes-Ferreira A., Oliveira J.M., Estevinho L.M., Mendes-Faia A. (2013). High-cell-density fermentation of *Saccharomyces cerevisiae* for the optimisation of mead production. Food Microbiol..

[17] Pereira A.P., Mendes-Ferreira A., Oliveira J.M., Estevinho L.M., Mendes-Faia A. (2014). Effect of *Saccharomyces cerevisiae* cells immobilisation on mead production. LWT-Food Sci. Technol..

[18] Roldán A., van Muiswinkel G.C.J., Lasanta C., Palacios V., Caro I. (2011). Influence of pollen addition on mead elaboration: Physicochemical and sensory characteristics. Food Chem..

[19] Kahoun D., Řezková S., Královský J. (2017). Effect of heat treatment and storage conditions on mead composition. Food Chem..

[20] Czabaj S., Kawa-Rygielska J., Kucharska A.Z., Kliks J. (2017). Effects of mead wort heat treatment on the mead fermentation process and antioxidant activity. Molecules.

[21] Kawa-Rygielska J., Adamenko K., Kucharska A.Z., Szatkowska K. (2019). Fruit and herbal meads–chemical composition and antioxidant properties. Food Chem..

[22] Šmogrovičová D., Nádaský P., Tandlich R., Wilhelmi B.S., Cambray G. (2012). Analytical and aroma profiles of Slovak and South African meads. Czech J. Food Sci..

[23] Sroka P., Tuszyński T. (2007). Changes in organic acid contents during mead wort fermentation. Food Chem..

[24] Ukpabi U.J. (2006). Quality evaluation of meads produced with Cassava (Manihot esculenta) floral honey under farm conditions in Nigeria. Trop. Subtrop. Agroecosyst..

[25] OIV—The International Organisation of Vine and Wine (2015). Compendium of International Methods of Wine and Must Analysis.

[26] Aerny J. (1996). Composés azotés des moûts et des vins. Rev. Suisse Vitic. Arboric. Hortic..

[27] Genisheva Z., Vilanova M., Mussatto S.I., Teixeira J.A., Oliveira J.M. (2014). Consecutive alcoholic fermentations of white grape musts with yeasts immobilized on grape skins-effect of biocatalyst storage and SO_2_ concentration on wine characteristic. LWT-Food Sci. Technol..

[28] Boidron J.N., Chatonnet P., Pons M. (1988). Influence du bois sur certaines substances odorantes des vins. Connaiss. J. Int. Sci. Vigne Vin.

[29] Escudero A., Gogorza B., Melús M.A., Ortín N., Cacho J., Ferreira V. (2004). Characterization of the aroma of a wine from Maccabeo. Key role played by compounds with low odor activity values. J. Agric. Food Chem..

[30] Ferreira V., López R., Cacho J.F. (2000). Quantitative determination of the odorants of young red wines from different grape varieties. J. Sci. Food Agric..

[31] Guth H. (1997). Quantification and sensory studies of character impact odorants of different white wine varieties. J. Agric. Food Chem..

[32] Moreno J.A., Zea L., Moyano L., Medina M. (2005). Aroma compounds as markers of the changes in sherry wines subjected to biological ageing. Food Control.

[33] Oliveira M.E.S., Pantoja L., Duarte W.F., Collela C.F., Valarelli L.T., Schwan R.F., Dias D.R. (2011). Fruit wine produced from cagaita (Eugenia dysenterica DC) by both free and immobilised yeast cell fermentation. Food Res. Int..

[34] Kassambara A., Mundt F. (2017). factoextra: Extract and Visualize the Results of Multivariate Data Analyses. http://www.sthda.com/english/rpkgs/factoextra.

[35] Ugliano M., Henschke P.A., Moreno-Arribas M.V., Polo M.C. (2009). Yeasts and wine flavour. Wine Chemistry and Biochemistry.

[36] Bartowsky E.J., Pretorius I.S., König H., Unden G., Fröhlich J. (2009). Microbial formation and modification of flavor and off-flavor compounds in wine. Biology of Microorganisms on Grapes, in Must and in Wine.

[37] Genisheva Z., Macedo S., Mussatto S.I., Teixeira J.A., Oliveira J.M. (2012). Production of white wine by *Saccharomyces cerevisiae* immobilized on grape pomace. J. Inst. Brew..

[38] Genisheva Z. (2013). Development of an integrated process for continuous winemaking. Ph.D. Thesis.

[39] Boulton B., Singleton V.L., Bisson L.F., Kunkee R.E. (1996). Principles and Practices of Winemaking.

[40] Tomás-Barberán F.A., Martos I., Ferreres F., Radovic B.S., Anklam E. (2001). HPLC flavonoid profiles as markers for the botanical origin of European unifloral honeys. J. Sci. Food Agric..

[41] Baumes R., Moreno-Arribas M.V., Polo M.C. (2009). Wine aroma precursors. Wine Chemistry and Biochemistry.

